# AT_2_ Receptor and Tissue Injury: Therapeutic Implications

**DOI:** 10.1007/s11906-013-0416-6

**Published:** 2014-01-11

**Authors:** Pawel Namsolleck, Chiara Recarti, Sébastien Foulquier, Ulrike Muscha Steckelings, Thomas Unger

**Affiliations:** 1 CARIM - School for Cardiovascular Diseases, Maastricht University, Maastricht, The Netherlands; 2 Institute of Molecular Medicine, Department of Cardiovascular and Renal Physiology, University of Southern Denmark, Odense, Denmark

**Keywords:** Renin-angiotensin system, AT_2_ receptor, Angiotensin II, CGP42112, PD123319, Signaling, Tissue injury, Inflammation, Fibrosis, Apoptosis, Growth promotion/inhibition, Cardioprotection, Vascular protection, Neuroprotection, Neuroregeneration, Myocardial infarction, Aortic aneurysm, Atherosclerosis, Hypertension, Stroke, Spinal cord injury

## Abstract

The renin-angiotensin system (RAS) plays an important role in the initiation and progression of tissue injuries in the cardiovascular and nervous systems. The detrimental actions of the AT_1_ receptor (AT_1_R) in hypertension and vascular injury, myocardial infarction and brain ischemia are well established. In the past twenty years, protective actions of the RAS, not only in the cardiovascular, but also in the nervous system, have been demonstrated. The so-called protective arm of the RAS includes AT_2_-receptors and Mas receptors (AT_2_R and MasR) and is characterized by effects different from and often opposing those of the AT_1_R. These include anti-inflammation, anti-fibrosis, anti-apoptosis and neuroregeneration that can counterbalance pathological processes and enable recovery from disease. The recent development of novel, small-molecule AT_2_R agonists offers a therapeutic potential in humans with a variety of clinical indications.

## Introduction

Research on the AT_2_ receptor (AT_2_R) started about 25 years ago with the discovery of two receptors for angiotensin II (Ang II), the AT_1_R and the AT_2_R. The AT_2_R was finally cloned in 1993 by two independent groups [1]. Due to its unusual properties including lack of the classic features of G-protein coupled receptor signaling, the AT_2_R was described as an “enigmatic” seven transmembrane receptor [2]. Research teams working on AT_2_R had to cope with several problems including a very selective tissue expression pattern in adult life and unusual, sometimes contradictory, physiological properties. Thus, the AT_2_R became “a matter of love and hate” [3]. Nowadays, it is well accepted that the AT_2_R forms part of the “protective arm of RAS” with a great potential in tissue protection and regeneration [4]. The AT_2_R, while being only sparsely expressed in most healthy tissues, is strongly upregulated following tissue damage [5] such as vascular [6] and neuronal injury [7], myocardial infarction (MI) [8–10] and brain ischemia [11]. The selective stimulation of the AT_2_R with a recently available small-molecule ligand, compound 21, has not only greatly helped to elucidate the major molecular pathways involved in AT_2_R-mediated tissue protection, such as anti-inflammation, anti-fibrosis and anti-apoptosis [5], but has also revealed a great potential for pharmacological intervention in the above-mentioned diseases [4]. This review summarizes current knowledge about the beneficial features of AT_2_R stimulation with restriction to cardiac, vascular and neuronal disease.

## AT_2_R-Mediated Signaling

AT_2_R stimulation activates at least three different classical signaling pathways: the cGMP/nitric oxide pathway [12, 13], protein phosphatases [14, 15] and phospholipase A2 signaling [16]. Since the tissue-protective properties of the AT_2_R are characterized mainly by regulation of inflammation, fibrosis and apoptosis [5], this review will focus on signaling pathways involved in these events.

### Inflammation

**Fig. 1:**
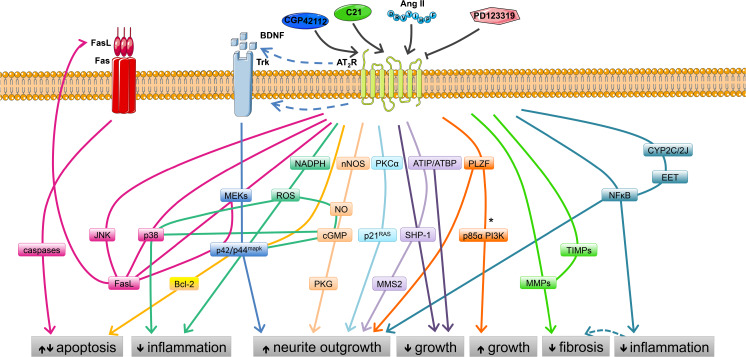
AT_2_R-mediated molecular pathways involved in tissue injury. The AT_2_R can be stimulated either by endogenous ligand Ang II or by exogenous agonists (CGP42112 or C21) resulting in activation or inhibition of multiple molecular pathways. This in turn leads to multiple cellular responses (grey boxes) including anti-inflammation, anti-fibrosis, modulation of apoptosis and enhanced neurite outgrowth. The MAPK-pathway and NFκB-pathway play central roles in the AT_2_R-mediated responses. Some of the molecular pathways are initiated indirectly via cross-talk with other receptors including Fas, TrkA and TrkB receptors by activation of the receptor or upregulation of ligand expression (e.g., FasL or BDNF). All pathways can be inhibited by the AT_2_R antagonist, PD123319, or by knocking-down the AT_2_R (not shown on the graph). Please notice that the edges in the pathway have undirected character (i.e., can represent either activation or inhibition). *growth factors are necessary for p85αPI3K activation. Abbreviations: *AT*_*2*_*R* angiotensin AT_2_ receptor; *Ang II* angiotensin II; *C21* compound 21 (non-peptide AT_2_R agonist); *CGP42112* peptide AT_2_R agonist; *PD123319* non-peptide AT_2_R antagonist; *Trk* tyrosine kinase receptor; *BDNF* brain-derived neurotrophic factor; *Fas* Fas cell surface death receptor; *FasL* Fas ligand; *CYP2C/2J* isoform of arachidonic acid-metabolizing cytochrome P450 enzyme; *EET* 11,12-epoxyeicosatrienoic acid; *NFκB* nuclear factor NF-kappa-B; *TIMP* tissue inhibitor of metalloproteinases; *MMPs* matrix metalloproteinases; *PKCα* protein kinase C, alpha; *p21* ^*RAS*^ Ras family of small GTP binding proteins; *ATIP/ATBP* AT_2_ receptor-interacting protein (or AT_2_ receptor binding protein); *SHP-1* protein-tyrosine phosphatase SHP-1; *MMS2* methyl methanesulfonate sensitive 2; *nNOS* neuronal nitric oxide synthase; *NO* nitric oxide; *cGMP* cyclic guanosine monophosphate; *PKG* cGMP-dependent protein kinase; *NADPH* reduced form of nicotinamide adenine dinucleotide phosphate; *ROS* reactive oxygen species; *MEK* mitogen-activated protein kinase kinase; *p42/p44* ^*mapk*^ p42/p44 mitogen-activated protein kinase; *Bcl-2* apoptosis regulator Bcl-2; *p38* mitogen-activated protein kinase p38; *JNK* JUN N-terminal kinase; *PLZF* promyelocytic leukemia zinc finger protein; *p85αPI3K* phosphatidylinositol 3-kinase regulatory subunit alpha

Key molecular mechanisms involved in the AT_2_R-mediated anti-inflammatory actions are the inhibition of NF-κB activity [17, 18] and the reduction of oxidative stress [19, 20] (Fig. 1).

In 2010, Rompe and colleagues demonstrated anti-inflammatory effects of AT_2_R stimulation via inhibition of cytokine levels in vitro and in vivo, using the orally active, highly selective, non-peptide AT_2_R agonist, compound 21 (C21). In this study, the authors showed that C21 caused a dose-dependent reduction of TNFα-induced interleukin-6 (Il-6) levels in primary human and murine dermal fibroblasts. Moreover, this study elucidated the anti-inflammatory AT_2_R-coupled signaling demonstrating that this pathway involves activation of protein phosphatases, CYP-dependent epoxidation of arachidonic acid to EETs, and inhibition of NF-κB activity. With this mechanism, AT_2_R counteracts not only the pro-inflammatory effects of TNFα but also those mediated by the AT_1_R, which involve CYP-dependent hydroxylation of arachidonic acid to 20-HETE and induces NF-κB activation [17].

As mentioned above, AT_2_R-mediated anti-inflammation can be achieved through an inhibition of oxidative stress. In fact, McCarthy et al., found that stimulation of the AT_2_R caused a reduction of stroke-induced superoxide production. They showed an inverse relationship between superoxide production and AT_2_R expression and suggested that AT_2_R reduces oxidative stress related to ischemia [19].

It is well known that Ang II induces oxidative stress via AT_1_R activation. Pendergrass et al., showed that AT_1_R-induced oxidative stress involves NADPH oxidase activation to generate reactive oxygen species (ROS) [21]. On the other hand, Dandapat and colleagues hypothesized that the AT_2_R is anti-inflammatory via reduction of pro-oxidant signals by inhibiting NADPH oxidase expression and ROS generation leading to a downregulation of p38 and p44/42 MAP kinase phosphorylation [22].

It is also known that, during oxidative stress, the production of ROS exceeds the available antioxidant defense systems. As a consequence, increased ROS concentrations reduce the amount of bioactive NO [23]. Moreover, it has been speculated that the signaling cascades activated by NO, including cGMP-dependent protein kinase activation, may be involved in downstream activation of mitogen-activated protein kinases that are required for IL-10 production [24]. In agreement with this hypothesis, Dhande and colleagues have recently demonstrated anti-inflammatory actions of the AT_2_R via increased interleukin-10 (IL-10) production in an NO-dependent manner [25].

In addition to the above-mentioned effects, increasing evidence demonstrates new protective anti-inflammatory actions of the AT_2_R via cellular mechanisms [26–29].

Curato and colleagues studied the role of the AT_2_R in the regulation of the cellular immune response in the context of ischemic heart injury. The authors identified a cardioprotective T cell population, CD8^+^AT_2_R^+^, characterized by upregulated IL-10 and downregulated IL-2 and INF-γ expression compared with CD8^+^AT_2_R^-^ T cells, which increased in response to ischemic cardiac injury. The authors demonstrated an immune-regulatory, cardioprotective action of the AT_2_R involving downregulation of the expression of proinflammatory cytokines and sustained IL-10 production, mediated, at least in part, *via* CD8^+^AT_2_R^+^ T cells [26].

Another recent study supports the immune regulatory role of the AT_2_R. Valero-Esquitino et al., evaluated the effects of AT_2_R stimulation on T cell differentiation in vitro. The authors concluded that AT_2_R stimulation induces an inhibition of T cells recruitment and modulation of the differentiation of naïve T cells into pro-inflammatory T helper (Th)1 and Th17 subsets while promoting differentiation into anti-inflammatory T regulatory cells [29].

### Fibrosis

Several investigators have observed anti-inflammatory actions of the AT_2_R concomitantly with anti-fibrosis suggesting a possible cross-talk between the two mechanisms [22, 30].

Moreover, different studies demonstrate that the anti-fibrotic activity of the AT_2_R seems to be due to a regulation of matrix metalloproteinases (MMP) and their inhibitors (TIMP) [22, 31–33] that play a key role in the regulation of the metabolic balance of the extracellular matrix (Fig. 1).

An important mechanism of AT_2_R-mediated anti-fibrosis appears to be an increased expression and activity of TIMP1 and TIMP2 with consequent inhibition of MMP9 and MMP2; however, the exact signaling pathway is still unknown.

Jing et al., showed in rat vascular smooth muscle cells (VSMCs) expressing the AT_2_R in a tetracyclin-regulated system, that the AT_2_R counteracted the effects elicited by AT_1_R signaling and caused a marked reduction in MMP2 levels [32]. In agreement with this finding, Brassard et al., demonstrated an AT_2_R-mediated decrease in MMP2, counteracting the AT_1_R-mediated increase in MMP2 activity and decrease in TIMP2 activity [31].

In a study investigating the role of the AT_2_R in atherosclerotic plaque, Dandapat et al., found that the AT_2_R reduced the expression and activity of MMP2 and MMP9, and collagen accumulation. The authors hypothesized that, like the anti-inflammatory pathway, this mechanism involves downregulation of NADPH oxidase and subsequent ROS generation leading to an inhibition of p38 MAPKS and p44/42 MAPKs phosphorylation [22].

In accordance with previous studies, an AT_2_R-mediated regulation of MMPs has been recently confirmed in a setting of atherosclerosis by Kljajic and colleagues. In this study, stimulation of AT_2_R, with the peptide AT_2_R agonist, CGP42112, caused a reduction of MMP2 and MMP9 [34].

Supporting the regulatory role of the AT_2_R in the TIMP/MMPs axis, Lauer et al., recently reported that in a rat model of infarct-induced heart failure, stimulation of the AT_2_R with the selective, non-peptide AT_2_R agonist, C21 induced an activation of TIMP1 and subsequent inhibition of MMP9-mediated proteolysis. Moreover, AT_2_R stimulation led to a downregulation of TGF-β1 followed by decreased collagen accumulation [33].

Conversely, Rehman et al., found that AT_2_R stimulation with C21 reduced fibrosis in stroke-prone spontaneously hypertensive rats without any difference in MMP2 and MMP9 expression [28]. A possible explanation for these contrasting results is that, in the latter study, only the expression and not the activity of MMPs and TIMPs was evaluated.

### Apoptosis

The role of the AT_2_R in apoptosis is controversial and seems to differ heavily depending on the experimental conditions (e.g., cell types, presence or absence of grow factors).

The AT_2_R regulates apoptosis via different pathways. AT_2_R stimulation can activate tyrosine phosphatases, such as mitogen-activated protein (MAP) kinase-phosphatase-1 (MKP-1) and inactivate MAP kinase extracellular signal-regulated kinase (ERK)1/2, resulting in dephosphorylation of Bcl-2 and upregulation of bax-induced pro-apoptotic effects [15, 35, 36] (Fig. 1).

Recently, we investigated the effect of AT_2_R stimulation by C21 on neuroprotection and neurite outgrowth and plasticity both in vitro and in vivo in a model of spinal cord injury in mice. This study reported improved neuronal survival with elevated expression of the neurotrophin, brain-derived neurotrophic factor (BDNF), and the neurotrophin receptors, TrkA and TrkB, as well as Bcl-2 that may link AT_2_R to anti-apoptosis [37].

Another pathway describing the AT_2_R-mediated regulation of apoptosis includes activation of caspases. Long-term stimulation of AT_2_R has been reported to cause ceramide generation leading to the activation of stress kinases and caspases and finally to apoptosis [38–40].

In fact, it has been reported in SMC that the AT_2_R mediates inducible transcriptional regulatory protein GATA-6 expression via activation of mitogen-activated protein kinase kinase (MEK)– ERK1/2 and c-Jun N-terminal kinase (JNK). GATA-6 in turn activates FasL promoter, FasL expression and consequently apoptosis via caspase 8 [41].

Conversely, different studies reported anti-apoptotic properties of the AT_2_R especially in pathological conditions. For instance, Kaschina et al., demonstrated that direct AT_2_R stimulation with C21 exerts beneficial effects after MI by anti-apoptotic and anti-inflammatory mechanisms. They showed that, stimulation of the AT_2_R engendered anti-apoptosis after MI by rescuing p38 MAPK and p44/42 MAPK expression and decreasing Fas-ligand and caspase-3 expression [30] (Fig. 1).

### Growth

It has been demonstrated that AT_2_R stimulation is anti-hypertrophic via two main mechanisms: activation of protein tyrosine phosphatase SHP-1, which blocks growth factor signals [42–44] and interaction with an AT_2_R binding protein (ATBP = ATIP) [45, 46]. A higher level of complexity has been added to the growth effects of the AT_2_R after the discovery of the promyelocytic leukemia zinc finger protein (PLZF) as an interacting protein of the AT_2_R [47], see below (Fig. 1).

### Neuronal Regeneration

The AT_2_R-related pathways in neuronal regeneration have been extensively studied in vitro in PC12W and NG108-15 cell-lines [48, 49]. AT_2_R-mediated neurite outgrowth seems to be a complex process that involves several pathways necessary for cytoskeleton rearrangements. In contrast to nerve growth factor, stimulation of the AT_2_R with Ang II leads to upregulation of beta-tubulin and MAP2 but downregulation of MAP1B [50] and neurofilament M [51] as shown in the PC12W cell-line. Signaling pathways involved in neurite outgrowth can be divided into four cascades that can be activated simultaneously or sequentially (Fig. 1). In the NG108-15 cell-line, stimulation of the AT_2_R leads to a decreased activity of PKCα followed by a decreased p21^RAS^ activity [52]. The second pathway involves phosphorylation of p42/p44^mapk^ mediated by Rap1/B-Raf [53]. However, this pathway may be initiated rather via the phosphorylation of tyrosine kinase receptor TrkA than directly downstream of the AT_2_R [54]. This is in agreement with our finding, where inhibition of tyrosine kinases with K252a abolished AT_2_R-mediated neurite outgrowth in primary cortical neurons [37]. However, the upregulation of TrkA, TrkB and BDNF suggest that autocrine and/or paracrine mechanisms may be involved as well. The third pathway is mediated via nitric oxide and cyclic GMP. Activation of nNOS via cGMP induces neurite elongation in NG108-15 cells [55]. Finally, the fourth cascade leading to neuronal differentiation involves ATIP (ATBP) [45, 46] and tyrosine phosphatase SHP-1 leading to transactivation of methyl methanesulfonate sensitive 2 enzyme (MMS2) [56, 57]. Other molecules such as PLZF or PPARγ may be involved in neurite outgrowth as well [56].

## AT_2_R in Cardiovascular Injury

While angiotensin AT_1_ receptor antagonists (ARBs) are well recognized and commonly used in the treatment of many cardiovascular disorders, the potential cardiovascular protection offered by the AT_2_R per se is less known. Direct AT_2_R stimulation has no impact on blood pressure; however, it seems to afford tissue/organ protection via anti-inflammatory and anti-fibrotic actions [58]. As reviewed previously, the expression of the AT_2_R is increased in different cardiovascular disorders such as left ventricular hypertrophy (LVH), heart failure, cardiac fibrosis and atherosclerosis [5, 59]. Here we review recent studies highlighting the cardiovascular protective actions and their related mechanisms induced by the AT_2_R.

### Cardioprotection

The pathophysiological role of AT_1_R stimulation in various kinds of heart disease is unanimously accepted. In particular, after myocardial infarction (MI), the AT_1_R contributes to tissue injury via inflammation and tissue remodeling. The AT_2_R, on the other hand, is widely recognized to induce actions counteracting those of the AT_1_R and this seems particularly true during cardiac injury [60].

Kaschina et al., performed the first study looking at the impact of direct AT_2_R stimulation post MI using the selective AT2R agonist, C21 [30]. In a model of MI following permanent coronary artery ligation in Wistar rats, acute treatment with C21 post-MI for one week improved cardiac function and decreased the infarct scar compared to vehicle treatment [30]. The underlying mechanisms seem to imply an anti-inflammatory as well as an anti-apoptotic action. Moreover, continuous AT_2_R stimulation can also afford long-term cardioprotection as highlighted recently in the late stage of MI six weeks after the acute event, when heart failure begins to develop [33]. C21 also improved arterial stiffness and reduced cardiac collagen content post-MI compared to the vehicle-treated group. The prevention of the cardiac remodeling by C21 seems to involve, among others, the TIMP1/MMP9 axis to reduce post-MI fibrosis [33].

In a model of MI with permanent ligature of the left anterior descending coronary artery, moderate cardio-selective overexpression of the AT_2_R also protected cardiac function and attenuated MI-induced cardiac hypertrophy [61]. The upregulation of the RAS components observed after MI in the left ventricle (mRNA) was attenuated in the presence of an overexpressed AT_2_R. Moreover, the MI-induced increase of collagen I was also attenuated [61]. In addition, other AT_2_R-mediated cardioprotective mechanisms, implying cardiac stem cells as well as CD8-positive T cells, have been identified [8, 26]. Indeed after MI, an increased AT_2_R expression in cardiac stem cells has been reported. Treatment of these cells with C21 attenuated the apoptosis of cardiomyocytes [8]. On the other hand, CD8^+^ AT_2_R^+^ T cells did not induce cytotoxicity to cardiomyocytes in opposition to the CD8^+^ AT_2_R^-^ T cells [26]. They also exhibited an anti-inflammatory pattern (see above). Moreover, intra-myocardial transplantation of CD8^+^ AT_2_R^+^ T cells after MI reduced the infarct size [26].

In a hypertension-induced model of left ventricular hypertrophy (SHR-SP), administration of C21, alone or in combination with an ARB, led to anti-fibrotic and anti-hypertrophic effects on the heart independently of any blood pressure change [28]. In particular, C21 reduced the interstitial collagen I/III content in the left ventricle and the expression of cardiac genes associated with cardiac hypertrophy. As suggested by the authors, this may be due to antioxidant and anti-inflammatory actions induced by AT_2_R stimulation [28]. Another recent study investigated the contribution of the AT_2_R to cardiac remodeling, which is commonly observed with chronic ARB treatment in the aging hypertensive heart [62]. In this study, cardiac fibrosis of aged SHR was reduced by AT_1_R blockade, and this cardioprotective effect was reversed by blockade of the AT_2_R, thus, highlighting the anti-fibrotic effects of the AT_2_R in the heart.

In a model of pulmonary hypertension, a disease that often leads to right ventricular failure due to the increased vasculature resistance in the lung, C21 treatment slowed the progression of the associated pulmonary and cardiac disease [63, 64], as observed by the reduction of right ventricular systolic pressure, right ventricular hypertrophy and dP/dtmax. These protective effects were prevented when the AT_2_R antagonist, PD123319, was administered concomitantly with C21. Moreover, C21 also attenuated the increase in pro-inflammatory cytokines contributing to the protective mechanisms of the AT_2_R stimulation in pulmonary hypertension [63, 64].

Cardioprotection afforded by the AT_2_R, thus, seems to be mainly the result of anti-inflammatory and anti-fibrotic actions. AT_2_R-mediated anti-inflammation is controlled by mechanisms initiated at two levels: within cardiac as well as within immune cells. This may offer new therapeutic perspectives against cardiac injuries.

In contrast to the results summarized above, the AT_2_R has also been considered as a mediator of cardiac hypertrophy. Infusion of Ang II in mice induces cardiac hypertrophy. However, in AT_2_R knock-out mice, cardiac hypertrophy is absent suggesting that the AT_2_R mediates this effect in response to increased blood pressure [65]. Further investigations suggested that the cardiac hypertrophic response mediated by the AT_2_R may involve the transcription factor PLZF [47, 66]. PLZF is able to bind the AT_2_R and recent in vivo studies confirmed its involvement in AT_2_R-mediated cardiac hypertrophy [67]. Following Ang II infusion, mice developed a major cardiac hypertrophy and fibrosis that was completely absent in PLZF knockout mice or in Ang II-infused wild type mice treated by an AT_2_R antagonist [67]. However, it should be noted at this point that the actions of PLZF depend on the growth factor environment for activation. This would indicate that the AT_2_R by itself does not induce cardiac hypertrophy, but that the recruitment of PLZF to the AT_2_R may, under appropriate conditions, switch the AT_2_R-mediated cardiac actions from antihypertrophic to hypertrophic ones [66].

### Vascular Protection

Using the L-NAME hypertension model that exhibits vascular remodeling with increased stiffness, Paulis et al., investigated the effect of chronic AT_2_R stimulation with C21 alone or in combination with the ARB olmesartan [68]. L-NAME treatment led to hypertension and hypertension-induced vascular changes characterized by an increased pulse wave velocity (PWV, an index of arterial stiffness), an increased aortic wall thickness, elastic modulus and aortic hydroxyproline content (marker for collagen deposition). C21 alone was able to partly prevent all these vascular injuries without any change in blood pressure. As expected, AT_1_R blockade with olmesartan completely prevented hypertension as well as the increase in PWV, aortic wall thickness and elastic modulus. Hydroxyproline accumulation in the aorta was slightly reduced by olmesartan, and the combination with C21 further reduced and even restored this parameter. Thus, the C21 + olmesartan combination led to a more pronounced stiffness reduction than each component alone, independently of any effect on blood pressure [68].

Similar mechanical/structural improvement of the vasculature after AT_2_R stimulation have also been reported in different vascular beds of SHR-SP (aorta, coronary arteries, and mesenteric arteries) [28]. Chronic administration of C21 alone or in combination with an ARB reduced vascular stiffness, collagen and fibronectin content. Moreover, C21 also improved the endothelial function as shown by the improved vasorelaxation to acetylcholine. These vasoprotective actions were observed concomitantly with a reduced superoxide production and monocyte/macrophage infiltration in the aorta [28].

The role of the AT_2_R has also been investigated in the pathological context of the Marfan syndrome, a disease that predisposes for aortic root aneurysm and aortic rupture. The authors crossed AT_2_R knock-out mice with a genetic mouse model of Marfan syndrome [69]. They observed a larger aortic root diameter in 2-month-old mice lacking the AT_2_R compared to mice with Marfan syndrome expressing the AT_2_R. This effect was maintained in one-year-old mice. In addition, they observed an increased death rate in AT_2_R KO mice with Marfan syndrome [69]. This highlights the key role of the AT_2_R in vascular remodeling since its absence accelerates the process of aortic aneurysm. The authors also investigated the contribution of indirect AT_2_R stimulation by using losartan in Marfan syndrome mice that express or not express the AT_2_R. They showed that AT_2_R signaling is needed to observe the protection afforded by losartan in this model [69].

In the pathological context of atherosclerosis, it has been previously suggested that the AT_2_R may play a role, since its absence increased the extent of the vascular lesions [70, 71]. This notion has been confirmed by a recent study using apoE-deficient transgenic mice overexpressing the AT_2_R [72]. The authors demonstrated that the AT_2_R-mediated anti-atherogenic actions were kinin / NO-dependent. Another recent study investigated the effect of direct AT_2_R stimulation using CGP42112 [34]. ApoE knock-out mice received a high fat diet for 16 weeks and were treated during the last four weeks with CGP42112. In this setting, AT_2_R stimulation showed vaso-protective and athero-protective effects with increased plaque stability. These effects were observed without any change on blood pressure and were associated with an improvement of the endothelial function and an increased NO bioavailability [34].

Although many studies have highlighted an AT_2_R-mediated vasoprotection, there are still some controversies. Opposed to the findings described above, a recent study showed that AT_2_R deficiency has no effect on either aortic aneurysms or atherosclerosis [73]. However the impact of the AT_2_R during tissue injuries should not be studied indirectly using AT_2_R blockade and/or AT_2_R knock-out animals but rather using direct stimulation of the AT_2_R.

## AT_2_R in Neuronal Injury

This chapter focuses on the AT_2_R-mediated neuroprotection and neuroregeneration. Neuroprotection can be defined as a process that directly prevents necrotic or apoptotic neuronal cell death (primary neuroprotection) or affords protection of myelin, axons, and neurons by, e.g., anti-inflammation (secondary neuroprotection). Neuroregeneration can be defined as a complex process restoring the interrupted neuronal connectivity and resulting in functional recovery. The underlying mechanisms may involve cell renewal, synaptic plasticity, regrowth of severed axons and sprouting of non-damaged neurons compensating the loss of a neighboring tract [74].

### Neuroprotection

Several studies have suggested a protective role of the AT_2_R in neuronal injury. The expression of AT_2_R is massively upregulated in neuronal damage as demonstrated in animal models of stroke and of sciatic or optic nerve crush [7, 11, 75]. Genetically modified animals lacking AT_2_R subjected to cerebral ischemia have larger infarcts as compared to the wild-type animals, mainly due to exacerbated inflammation and generation of ROS [76]. Subsequent studies demonstrated that stimulation of the AT_2_R exerts neuroprotection either directly, or by the anti-inflammatory activity (secondary neuroprotection).

The neuro-protective potential of AT_2_R-stimulation was demonstrated in vitro in *N*-methyl-d-aspartate (NMDA)-mediated cell death [77]. The survival of two differentiated neuronal cell lines, NG108-15 and N1E115, was reduced by treatment with NMDA in a dose-dependent manner. Incubation with 10^-7^ M Ang II inhibited neuronal apoptosis and suppressed the NMDA-mediated reduction of anti-apoptotic Bcl-2. This effect was inhibited by the co-treatment with PD123319, but not with losartan, suggesting an AT_2_R-mediated primary neuroprotection [77]. In primary cortical neurons isolated from mouse embryos, AT_2_R stimulation with CGP42112 significantly reduced cell death during glucose deprivation [78]. This effect was blocked by the co-treatment with PD123319 confirming the AT_2_R specificity.

Postischemic inflammation significantly contributes to ischemic brain injury. Therefore, anti-inflammatory therapeutic strategies can afford secondary neuroprotection in ischemic stroke [79]. Previously, we demonstrated that AT_2_R stimulation inhibits inflammatory response in vitro in CNS-derived cells. As shown in primary rat astrocytes exposed to LPS, the elevated expression of IL-6 and TNF-α was significantly reduced by treatment with C21 as compared to vehicle [80]. AT_2_R stimulation also inhibited microglial activation as shown by reduced nitric oxide release, and abolished cell migration [81].

In animal models of neuronal injury, both primary and secondary neuroprotection may significantly contribute to the beneficial effects mediated by the AT_2_R. Previously, we had shown that the AT_1_R blocker telmisartan, but not the ACE inhibitor ramipril, reduced infarct volume, neuronal apoptosis and inflammation in normotensive rats subjected to middle cerebral artery occlusion (MCAO) [82]. This observation may suggest that, in the presence of AT_1_R blockade, endogenous Ang II stimulates the unopposed AT_2_R and by this exerts neuroprotective actions.

The AT_2_R-mediated neuroprotection in cerebral ischemia was extensively studied by another group [19, 78, 83]. In spontaneously hypertensive rats, intracerebroventricular (i.c.v.) administration of CGP42112 for five days prior to endothelin-1 induced stroke reduced infarct volume, locomotor deficits and neuronal apoptosis as measured 72 hours after cerebral ischemia [19]. Importantly, stimulation of the AT_2_R even six hours after stroke induction was still effective, showing for the first time a neuroprotective effect of delayed AT_2_R stimulation after cerebral ischemia [83]. In C57Bl6 mice subjected to MCAO, systemic administration of the peptidic AT_2_R agonist, CGP42112, during the reperfusion reduced infarct and edema volume as well as improved functional outcome [78]. It is unclear, whether CGP42112 can enter brain parenchyma through the damaged blood-brain barrier. However, improved cerebral blood flow during the first ten minutes of reperfusion suggests, that vascular components may contribute to the observed beneficial effects of systemic drug administration [78].

Our group studied the AT_2_R-mediated neuroprotective mechanisms in MCAO in mice [84]. Systemic administration of C21 reduced post-stroke mortality, neurological deficits and neuronal apoptosis. This was accompanied by a reduced IL-6 expression and elevated BDNF in the infarcted brain areas [84]. These findings suggest that the neurotrophic pathway is involved in the observed neuroprotection by promoting neuronal survival.

### Neuroregeneration

Neurite outgrowth has been attributed to the AT_2_R [85, 86] indicating a neuroregenerative potential [1]. In primary cortical neurons isolated from embryonic rat, Ang II promoted neurite outgrowth as estimated by the measurement of neurite length. Treatment with irbesartan enhanced the Ang II-mediated effect, and PD123319 completely abolished neurite outgrowth [11]. Similar effects were obtained when the AT_2_R was stimulated directly; either with CGP42112 [85] or with C21 [37, 87].

Lucius et al., demonstrated already in 1998 that AT_2_R stimulation engendered neurotrophic-like actions in the nervous system of adult animals [75]. Rats subjected to the optic nerve lesion and treated locally with Ang II showed outgrowth of axon bundles within the proximal optic nerve. This effect was AT_2_R-dependent since it could be inhibited by an AT_2_R-antagonist [75]. The neuroregenerative potential of AT_2_R was also demonstrated in a sciatic nerve crush model in rats [88]. Ang II not only increased axonal diameters and promoted re-myelination via AT_2_R but also accelerated functional recovery as shown by increased toe spread distance (parameter for motor-function) and improved the foot reflex withdrawal reaction (a parameter of sensomotoric function) [88]. Finally, in rats subjected to MCAO, increased AT_2_R immunoreactivity was associated with intense neurite outgrowth in ischemic striatum [11].

The AT_2_R may be implied in neuro-regenerative processes beyond the well-described neurite outgrowth. It has been shown that in AT_2_R-KO mice subjected to MCAO, impaired spatial memory was associated with decreased hippocampal neurogenesis as compared to wild-type animals [89]. This effect was larger in female animals, suggesting gender differences in AT_2_R-mediated neuroregeneration. In a related manner, the impact of AT_2_R-stimulation on renal function also differs between sexes in that direct AT_2_R-stimulation with C21 increased renal blood flow to a greater extent in female rats than in males [90].

We have recently evaluated the impact of the direct AT_2_R stimulation with C21 on neuroregeneration in an animal model of spinal cord injury (SCI) [37]. In mice subjected to SCI, treatment with C21 elevated the number of regenerating axons cranially and caudally from the lesioned area. The number of regenerating fibers positively correlated with improved locomotor performance indicating functionality of these fibers. Animals treated with C21 showed elevated immunoreactivity for the neutrophic receptor TrkB in the peri-lesional area. Parallel in vitro studies confirmed the importance of the BDNF/TrkB axis in AT_2_R-mediated neuroregeneration [37].

Apart from neuronal injury, the exogenous delivery of BDNF to the CNS may exert therapeutic effects in a variety of other neurological diseases including Alzheimer’s, Parkinson’s and Huntington’s disease, amyotrophic lateral sclerosis, Rett syndrome and vascular dementia [91, 92]. However, gene delivery in humans remains a challenge. On the other hand, a drug-induced increase of endogenous BDNF expression in the CNS seems to be a rational alternative for gene therapy. Small molecule AT_2_R agonists, such as C21, via the activation of neutrophic pathways, may provide a therapeutic option in the above-mentioned diseases.

## Conclusions

During the twenty-five years following the discovery of the AT_2_R, our understanding of its protective role in tissue injury has greatly improved, but it is still far from complete. Development of pharmacological tools such as small-molecule ligands and the generation of the AT_2_R-KO animals have facilitated the elucidation of the major molecular pathways engaged by the AT_2_R. Results from various experimental disease models revealed the protective and regenerative potential of AT_2_R stimulation in cardiovascular and neuronal injury. Future research on the AT_2_R should concentrate on the complexity of molecular AT_2_R-related pathways and the cross-talk with other receptors and pathways in the context of tissue-protection. In addition to basic science, translational research will have to address the therapeutic potential of AT_2_R stimulation with available AT_2_R agonists in humans and define therapeutic cardiovascular indications and non-cardiovascular indications.
